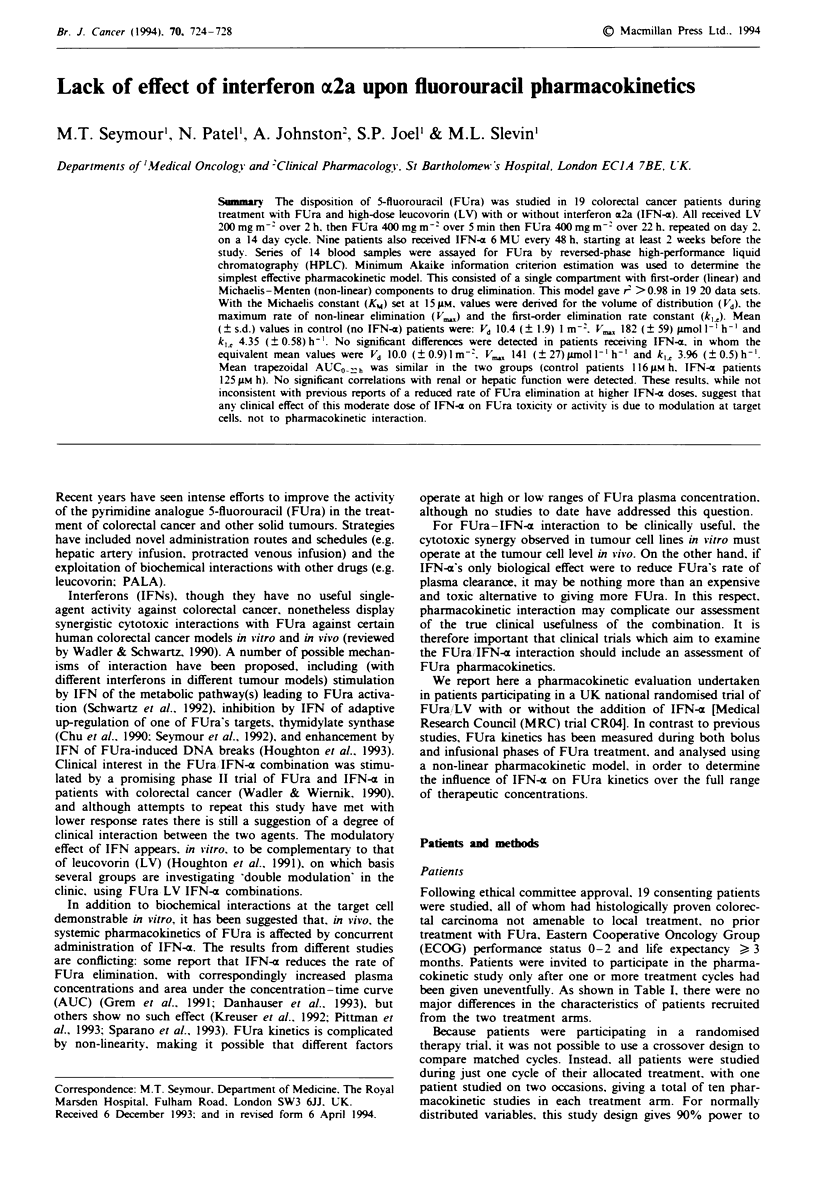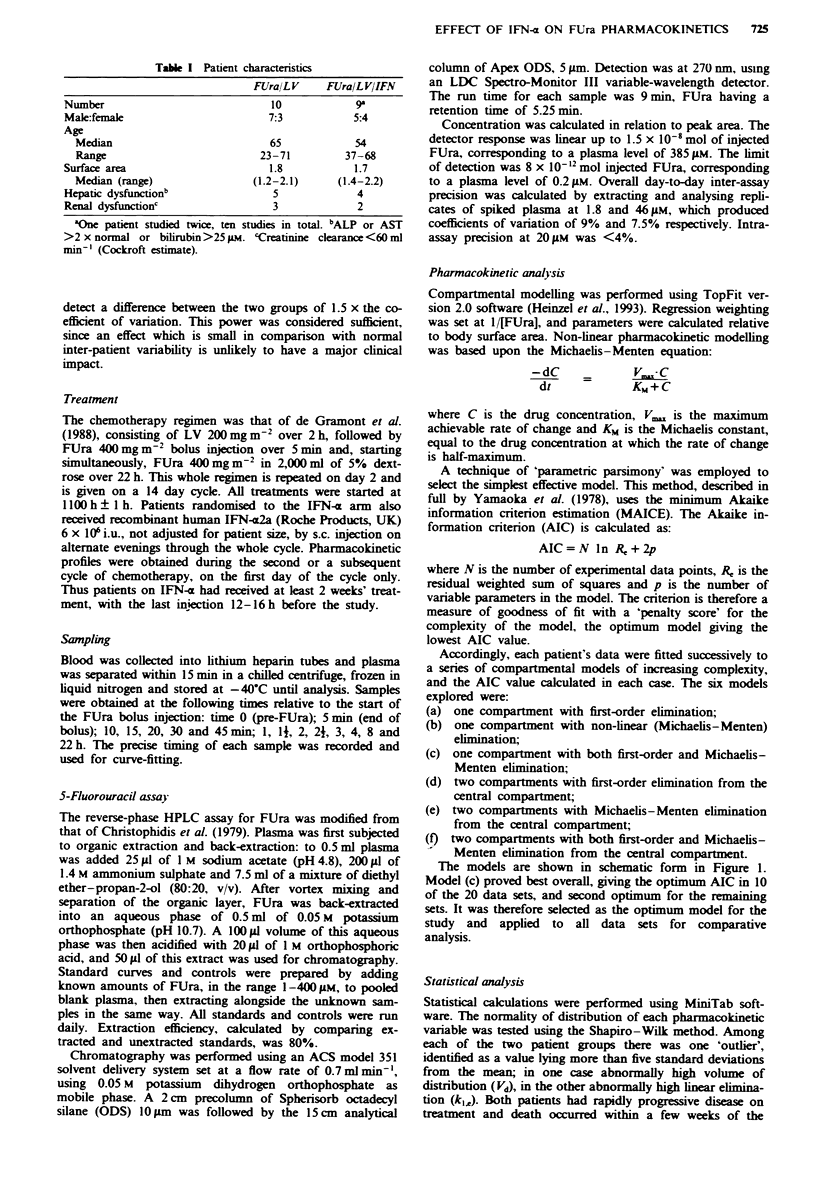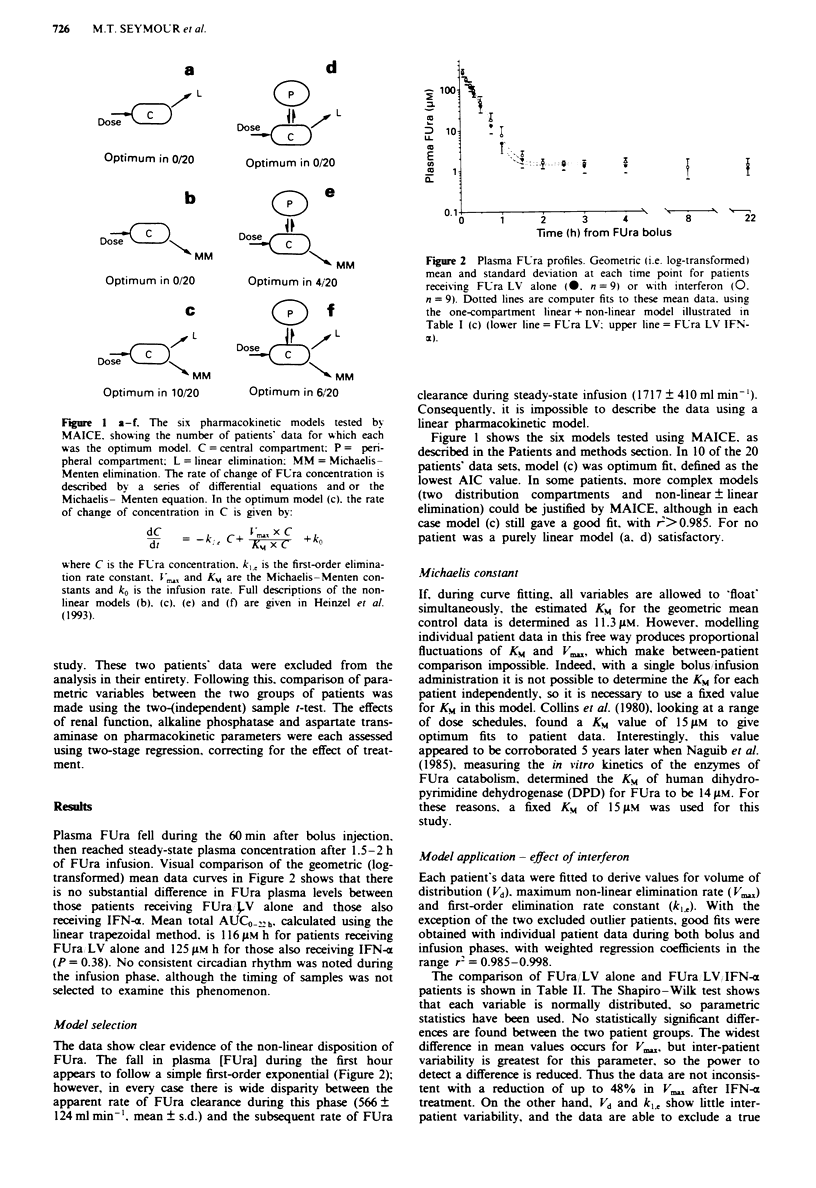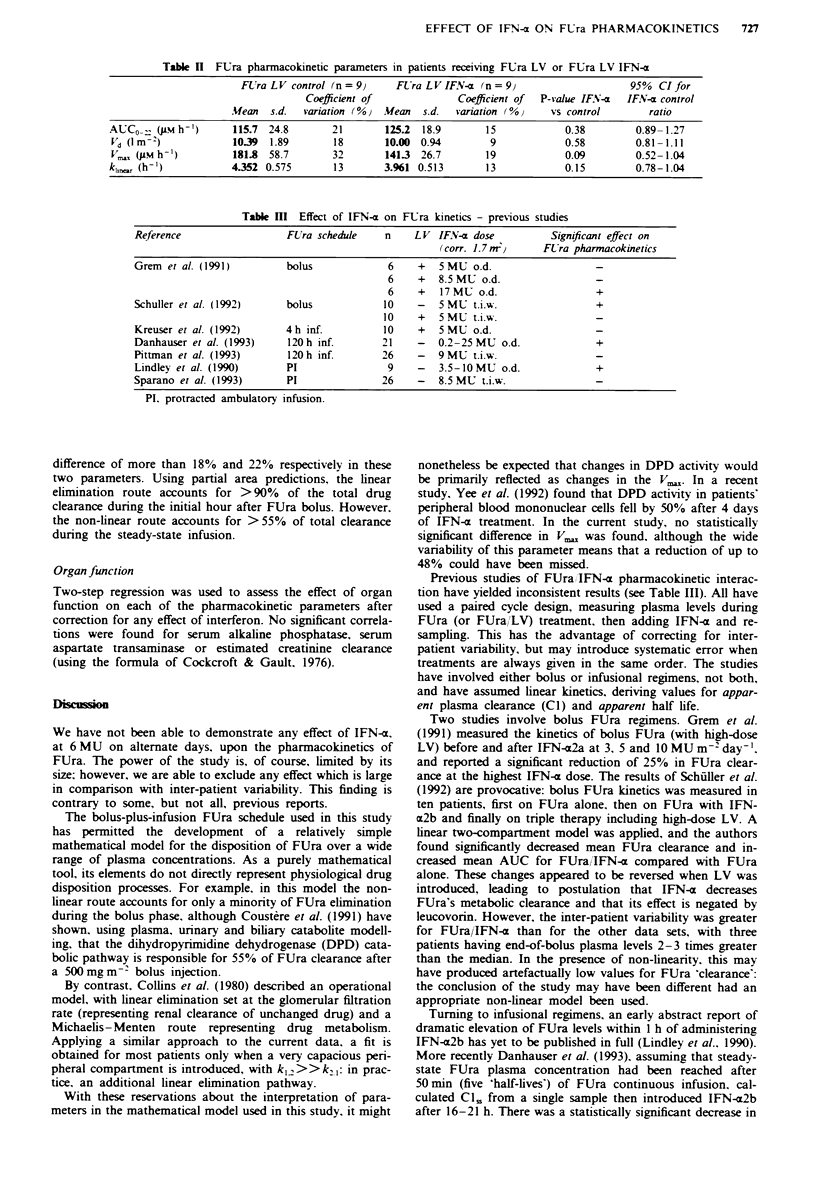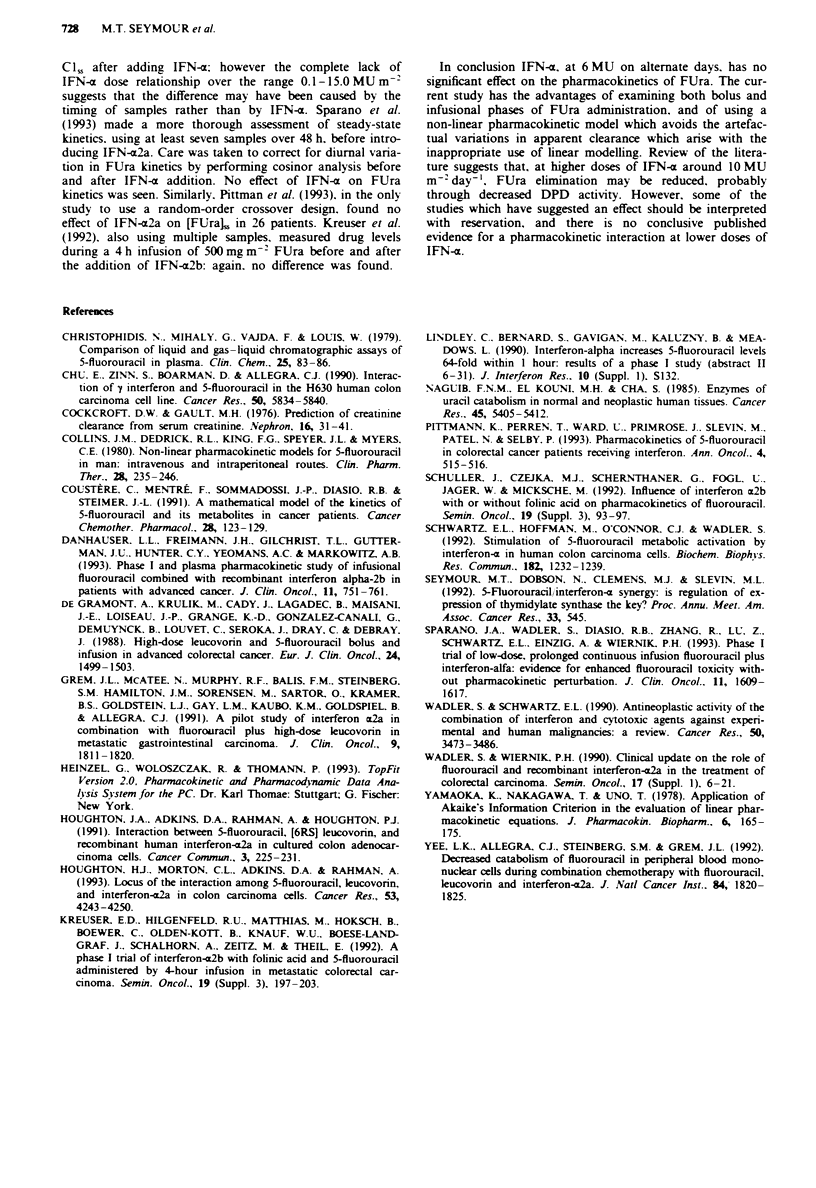# Lack of effect of interferon alpha 2a upon fluorouracil pharmacokinetics.

**DOI:** 10.1038/bjc.1994.383

**Published:** 1994-10

**Authors:** M. T. Seymour, N. Patel, A. Johnston, S. P. Joel, M. L. Slevin

**Affiliations:** Department of Medical Oncology, St Bartholomew's Hospital, London, UK.

## Abstract

The disposition of 5-fluorouracil (FUra) was studied in 19 colorectal cancer patients during treatment with FUra and high-dose leucovorin (LV) with or without interferon alpha 2a (IFN-alpha). All received LV 200 mg m-2 over 2 h, then FUra 400 mg m-2 over 5 min then FUra 400 mg m-2 over 22 h, repeated on day 2, on a 14 day cycle. Nine patients also received IFN-alpha 6 MU every 48 h, starting at least 2 weeks before the study. Series of 14 blood samples were assayed for FUra by reversed-phase high-performance liquid chromatography (HPLC). Minimum Akaike information criterion estimation was used to determine the simplest effective pharmacokinetic model. This consisted of a single compartment with first-order (linear) and Michaelis-Menten (non-linear) components to drug elimination. This model gave r2 > 0.98 in 19/20 data sets. With the Michaelis constant (KM) set at 15 microM, values were derived for the volume of distribution (Vd), the maximum rate of non-linear elimination (Vmax) and the first-order elimination rate constant (K1.e). Mean (+/- s.d.) values in control (no IFN-alpha) patients were: Vd 10.4 (+/- 1.9) l m-2, Vmax 182 (+/- 59) mumol l-1 h-1 and k1.e 4.35 (+/- 0.58) h-1. No significant differences were detected in patients receiving IFN-alpha, in whom the equivalent mean values were Vd 10.0 (+/- 0.9) l m-2, Vmax 141 (+/- 27) mumol l-1 h-1 and k1.e 3.96 (+/- 0.5) h-1. Mean trapezoidal AUC0-22 h was similar in the two groups (control patients 116 microM h, IFN-alpha patients 125 microM h). No significant correlations with renal or hepatic function were detected. These results, while not inconsistent with previous reports of a reduced rate of FUra elimination at higher IFN-alpha doses, suggest that any clinical effect of this moderate dose of IFN-alpha on FUra toxicity or activity is due to modulation at target cells, not to pharmacokinetic interaction.


 © Macmillan Press Ltd., 1994

### Lack of effect of interferon α2a upon fluorouracil pharmacokinetics

M.T. Seymour[1], N. Patel[1], A. Johnston[2], S.P. Joel[1] & M.L. Slevin[1]

*Departments of [1]Medical Oncology and [2]Clinical Pharmacology, St Bartholomew's Hospital, London EC1A 7BE, UK.*

**Summary** The disposition of 5-fluorouracil (FUra) was studied in 19 colorectal cancer patients during treatment with FUra and high-dose leucovorin (LV) with or without interferon α2a (IFN-α). All received LV 200 mg m$^{-2}$ over 2 h, then FUra 400 mg m$^{-2}$ over 5 min then FUra 400 mg m$^{-2}$ over 22 h, repeated on day 2, on a 14 day cycle. Nine patients also received IFN-α 6 MU every 48 h, starting at least 2 weeks before the study. Series of 14 blood samples were assayed for FUra by reversed-phase high-performance liquid chromatography (HPLC). Minimum Akaike information criterion estimation was used to determine the simplest effective pharmacokinetic model. This consisted of a single compartment with first-order (linear) and Michaelis-Menten (non-linear) components to drug elimination. This model gave $r^2 > 0.98$ in 19/20 data sets. With the Michaelis constant ($K_M$) set at 15 μM, values were derived for the volume of distribution ($V_d$), the maximum rate of non-linear elimination ($V_{max}$) and the first-order elimination rate constant ($k_{1,e}$). Mean (± s.d.) values in control (no IFN-α) patients were: $V_d$ 10.4 (± 1.9) l m$^{-2}$, $V_{max}$ 182 (± 59) μmol l$^{-1}$ h$^{-1}$ and $k_{1,e}$ 4.35 (± 0.58) h$^{-1}$. No significant differences were detected in patients receiving IFN-α, in whom the equivalent mean values were $V_d$ 10.0 (± 0.9) l m$^{-2}$, $V_{max}$ 141 (± 27) μmol l$^{-1}$ h$^{-1}$ and $k_{1,e}$ 3.96 (± 0.5) h$^{-1}$. Mean trapezoidal $AUC_{0-22h}$ was similar in the two groups (control patients 116 μM h, IFN-α patients 125 μM h). No significant correlations with renal or hepatic function were detected. These results, while not inconsistent with previous reports of a reduced rate of FUra elimination at higher IFN-α doses, suggest that any clinical effect of this moderate dose of IFN-α on FUra toxicity or activity is due to modulation at target cells, not to pharmacokinetic interaction.

Recent years have seen intense efforts to improve the activity of the pyrimidine analogue 5-fluorouracil (FUra) in the treatment of colorectal cancer and other solid tumours. Strategies have included novel administration routes and schedules (e.g. hepatic artery infusion, protracted venous infusion) and the exploitation of biochemical interactions with other drugs (e.g. leucovorin; PALA).

Interferons (IFNs), though they have no useful single-agent activity against colorectal cancer, nonetheless display synergistic cytotoxic interactions with FUra against certain human colorectal cancer models *in vitro* and *in vivo* (reviewed by Wadler & Schwartz, 1990). A number of possible mechanisms of interaction have been proposed, including (with different interferons in different tumour models) stimulation by IFN of the metabolic pathway(s) leading to FUra activation (Schwartz *et al.*, 1992), inhibition by IFN of adaptive up-regulation of one of FUra's targets, thymidylate synthase (Chu *et al.*, 1990; Seymour *et al.*, 1992), and enhancement by IFN of FUra-induced DNA breaks (Houghton *et al.*, 1993). Clinical interest in the FUra/IFN-α combination was stimulated by a promising phase II trial of FUra and IFN-α in patients with colorectal cancer (Wadler & Wiernik, 1990), and although attempts to repeat this study have met with lower response rates there is still a suggestion of a degree of clinical interaction between the two agents. The modulatory effect of IFN appears, *in vitro*, to be complementary to that of leucovorin (LV) (Houghton *et al.*, 1991), on which basis several groups are investigating 'double modulation' in the clinic, using FUra/LV/IFN-α combinations.

In addition to biochemical interactions at the target cell demonstrable *in vitro*, it has been suggested that, *in vivo*, the systemic pharmacokinetics of FUra is affected by concurrent administration of IFN-α. The results from different studies are conflicting: some report that IFN-α reduces the rate of FUra elimination, with correspondingly increased plasma concentrations and area under the concentration-time curve (AUC) (Grem *et al.*, 1991; Danhauser *et al.*, 1993), but others show no such effect (Kreuser *et al.*, 1992; Pittman *et al.*, 1993; Sparano *et al.*, 1993). FUra kinetics is complicated by non-linearity, making it possible that different factors operate at high or low ranges of FUra plasma concentration, although no studies to date have addressed this question.

For FUra-IFN-α interaction to be clinically useful, the cytotoxic synergy observed in tumour cell lines *in vitro* must operate at the tumour cell level *in vivo*. On the other hand, if IFN-α's only biological effect were to reduce FUra's rate of plasma clearance, it may be nothing more than an expensive and toxic alternative to giving more FUra. In this respect, pharmacokinetic interaction may complicate our assessment of the true clinical usefulness of the combination. It is therefore important that clinical trials which aim to examine the FUra/IFN-α interaction should include an assessment of FUra pharmacokinetics.

We report here a pharmacokinetic evaluation undertaken in patients participating in a UK national randomised trial of FUra/LV with or without the addition of IFN-α [Medical Research Council (MRC) trial CR04]. In contrast to previous studies, FUra kinetics has been measured during both bolus and infusional phases of FUra treatment, and analysed using a non-linear pharmacokinetic model, in order to determine the influence of IFN-α on FUra kinetics over the full range of therapeutic concentrations.

### Patients and methods

### *Patients*

Following ethical committee approval, 19 consenting patients were studied, all of whom had histologically proven colorectal carcinoma not amenable to local treatment, no prior treatment with FUra, Eastern Cooperative Oncology Group (ECOG) performance status 0-2 and life expectancy ≥ 3 months. Patients were invited to participate in the pharmacokinetic study only after one or more treatment cycles had been given uneventfully. As shown in Table I, there were no major differences in the characteristics of patients recruited from the two treatment arms.

Because patients were participating in a randomised therapy trial, it was not possible to use a crossover design to compare matched cycles. Instead, all patients were studied during just one cycle of their allocated treatment, with one patient studied on two occasions, giving a total of ten pharmacokinetic studies in each treatment arm. For normally distributed variables, this study design gives 90% power to

Correspondence: M.T. Seymour, Department of Medicine, The Royal Marsden Hospital, Fulham Road, London SW3 6JJ, UK.
Received 6 December 1993; and in revised form 6 April 1994.

**Table I** Patient characteristics

| | *FUra/LV* | *FUra/LV/IFN* |
|---|---|---|
| Number | 10 | 9[a] |
| Male:female | 7:3 | 5:4 |
| Age | | |
| Median | 65 | 54 |
| Range | 23–71 | 37–68 |
| Surface area | 1.8 | 1.7 |
| Median (range) | (1.2–2.1) | (1.4–2.2) |
| Hepatic dysfunction[b] | 5 | 4 |
| Renal dysfunction[c] | 3 | 2 |

[a]One patient studied twice, ten studies in total. [b]ALP or AST >2 × normal or bilirubin>25 μM. [c]Creatinine clearance<60 ml $min^{-1}$ (Cockroft estimate).

detect a difference between the two groups of 1.5 × the coefficient of variation. This power was considered sufficient, since an effect which is small in comparison with normal inter-patient variability is unlikely to have a major clinical impact.

### *Treatment*

The chemotherapy regimen was that of de Gramont *et al.* (1988), consisting of LV 200 mg $m^{-2}$ over 2 h, followed by FUra 400 mg $m^{-2}$ bolus injection over 5 min and, starting simultaneously, FUra 400 mg $m^{-2}$ in 2,000 ml of 5% dextrose over 22 h. This whole regimen is repeated on day 2 and is given on a 14 day cycle. All treatments were started at 1100 h ± 1 h. Patients randomised to the IFN-α arm also received recombinant human IFN-α2a (Roche Products, UK) 6 × $10^6$ i.u., not adjusted for patient size, by s.c. injection on alternate evenings through the whole cycle. Pharmacokinetic profiles were obtained during the second or a subsequent cycle of chemotherapy, on the first day of the cycle only. Thus patients on IFN-α had received at least 2 weeks' treatment, with the last injection 12–16 h before the study.

### *Sampling*

Blood was collected into lithium heparin tubes and plasma was separated within 15 min in a chilled centrifuge, frozen in liquid nitrogen and stored at −40°C until analysis. Samples were obtained at the following times relative to the start of the FUra bolus injection: time 0 (pre-FUra); 5 min (end of bolus); 10, 15, 20, 30 and 45 min; 1, 1½, 2, 2½, 3, 4, 8 and 22 h. The precise timing of each sample was recorded and used for curve-fitting.

### *5-Fluorouracil assay*

The reverse-phase HPLC assay for FUra was modified from that of Christophidis *et al.* (1979). Plasma was first subjected to organic extraction and back-extraction: to 0.5 ml plasma was added 25 μl of 1 M sodium acetate (pH 4.8), 200 μl of 1.4 M ammonium sulphate and 7.5 ml of a mixture of diethyl ether–propan-2-ol (80:20, v/v). After vortex mixing and separation of the organic layer, FUra was back-extracted into an aqueous phase of 0.5 ml of 0.05 M potassium orthophosphate (pH 10.7). A 100 μl volume of this aqueous phase was then acidified with 20 μl of 1 M orthophosphoric acid, and 50 μl of this extract was used for chromatography. Standard curves and controls were prepared by adding known amounts of FUra, in the range 1–400 μM, to pooled blank plasma, then extracting alongside the unknown samples in the same way. All standards and controls were run daily. Extraction efficiency, calculated by comparing extracted and unextracted standards, was 80%.

Chromatography was performed using an ACS model 351 solvent delivery system set at a flow rate of 0.7 ml $min^{-1}$, using 0.05 M potassium dihydrogen orthophosphate as mobile phase. A 2 cm precolumn of Spherisorb octadecyl silane (ODS) 10 μm was followed by the 15 cm analytical column of Apex ODS, 5 μm. Detection was at 270 nm, using an LDC Spectro-Monitor III variable-wavelength detector. The run time for each sample was 9 min, FUra having a retention time of 5.25 min.

Concentration was calculated in relation to peak area. The detector response was linear up to 1.5 × $10^{-8}$ mol of injected FUra, corresponding to a plasma level of 385 μM. The limit of detection was 8 × $10^{-12}$ mol injected FUra, corresponding to a plasma level of 0.2 μM. Overall day-to-day inter-assay precision was calculated by extracting and analysing replicates of spiked plasma at 1.8 and 46 μM, which produced coefficients of variation of 9% and 7.5% respectively. Intra-assay precision at 20 μM was <4%.

### *Pharmacokinetic analysis*

Compartmental modelling was performed using TopFit version 2.0 software (Heinzel *et al.*, 1993). Regression weighting was set at 1/[FUra], and parameters were calculated relative to body surface area. Non-linear pharmacokinetic modelling was based upon the Michaelis–Menten equation:

$$\frac{-dC}{dt} = \frac{V_{max} \cdot C}{K_M + C}$$

where $C$ is the drug concentration, $V_{max}$ is the maximum achievable rate of change and $K_M$ is the Michaelis constant, equal to the drug concentration at which the rate of change is half-maximum.

A technique of 'parametric parsimony' was employed to select the simplest effective model. This method, described in full by Yamaoka *et al.* (1978), uses the minimum Akaike information criterion estimation (MAICE). The Akaike information criterion (AIC) is calculated as:

$$AIC = N \ln R_e + 2p$$

where $N$ is the number of experimental data points, $R_e$ is the residual weighted sum of squares and $p$ is the number of variable parameters in the model. The criterion is therefore a measure of goodness of fit with a 'penalty score' for the complexity of the model, the optimum model giving the lowest AIC value.

Accordingly, each patient's data were fitted successively to a series of compartmental models of increasing complexity, and the AIC value calculated in each case. The six models explored were:

(a) one compartment with first-order elimination;
(b) one compartment with non-linear (Michaelis–Menten) elimination;
(c) one compartment with both first-order and Michaelis–Menten elimination;
(d) two compartments with first-order elimination from the central compartment;
(e) two compartments with Michaelis–Menten elimination from the central compartment;
(f) two compartments with both first-order and Michaelis–Menten elimination from the central compartment.

The models are shown in schematic form in Figure 1. Model (c) proved best overall, giving the optimum AIC in 10 of the 20 data sets, and second optimum for the remaining sets. It was therefore selected as the optimum model for the study and applied to all data sets for comparative analysis.

### *Statistical analysis*

Statistical calculations were performed using MiniTab software. The normality of distribution of each pharmacokinetic variable was tested using the Shapiro–Wilk method. Among each of the two patient groups there was one 'outlier', identified as a value lying more than five standard deviations from the mean; in one case abnormally high volume of distribution ($V_d$), in the other abnormally high linear elimination ($k_{1,e}$). Both patients had rapidly progressive disease on treatment and death occurred within a few weeks of the

**Figure 1** a–f. The six pharmacokinetic models tested by MAICE, showing the number of patients' data for which each was the optimum model. C = central compartment; P = peripheral compartment; L = linear elimination; MM = Michaelis–Menten elimination. The rate of change of FUra concentration is described by a series of differential equations and/or the Michaelis–Menten equation. In the optimum model (c), the rate of change of concentration in C is given by:

$$\frac{dC}{dt} = -k_{l,e}\,C + \frac{V_{max} \times C}{K_M \times C} + k_0$$

where $C$ is the FUra concentration, $k_{1,e}$ is the first-order elimination rate constant, $V_{max}$ and $K_M$ are the Michaelis–Menten constants and $k_0$ is the infusion rate. Full descriptions of the non-linear models (b), (c), (e) and (f) are given in Heinzel et al. (1993).

**Figure 2** Plasma FUra profiles. Geometric (i.e. log-transformed) mean and standard deviation at each time point for patients receiving FUra/LV alone (●, $n = 9$) or with interferon (O, $n = 9$). Dotted lines are computer fits to these mean data, using the one-compartment linear + non-linear model illustrated in Table I (c) (lower line = FUra/LV; upper line = FUra/LV/IFN-α).

study. These two patients' data were excluded from the analysis in their entirety. Following this, comparison of parametric variables between the two groups of patients was made using the two-(independent) sample $t$-test. The effects of renal function, alkaline phosphatase and aspartate transaminase on pharmacokinetic parameters were each assessed using two-stage regression, correcting for the effect of treatment.

### Results

Plasma FUra fell during the 60 min after bolus injection, then reached steady-state plasma concentration after 1.5–2 h of FUra infusion. Visual comparison of the geometric (log-transformed) mean data curves in Figure 2 shows that there is no substantial difference in FUra plasma levels between those patients receiving FUra/LV alone and those also receiving IFN-α. Mean total $AUC_{0-22\,h}$, calculated using the linear trapezoidal method, is 116 μM h for patients receiving FUra/LV alone and 125 μM h for those also receiving IFN-α ($P = 0.38$). No consistent circadian rhythm was noted during the infusion phase, although the timing of samples was not selected to examine this phenomenon.

### *Model selection*

The data show clear evidence of the non-linear disposition of FUra. The fall in plasma [FUra] during the first hour appears to follow a simple first-order exponential (Figure 2); however, in every case there is wide disparity between the apparent rate of FUra clearance during this phase (566 ± 124 ml $min^{-1}$, mean ± s.d.) and the subsequent rate of FUra clearance during steady-state infusion (1717 ± 410 ml $min^{-1}$). Consequently, it is impossible to describe the data using a linear pharmacokinetic model.

Figure 1 shows the six models tested using MAICE, as described in the Patients and methods section. In 10 of the 20 patients' data sets, model (c) was optimum fit, defined as the lowest AIC value. In some patients, more complex models (two distribution compartments and non-linear ± linear elimination) could be justified by MAICE, although in each case model (c) still gave a good fit, with $r^2 > 0.985$. For no patient was a purely linear model (a, d) satisfactory.

### *Michaelis constant*

If, during curve fitting, all variables are allowed to 'float' simultaneously, the estimated $K_M$ for the geometric mean control data is determined as 11.3 μM. However, modelling individual patient data in this free way produces proportional fluctuations of $K_M$ and $V_{max}$, which make between-patient comparison impossible. Indeed, with a single bolus/infusion administration it is not possible to determine the $K_M$ for each patient independently, so it is necessary to use a fixed value for $K_M$ in this model. Collins et al. (1980), looking at a range of dose schedules, found a $K_M$ value of 15 μM to give optimum fits to patient data. Interestingly, this value appeared to be corroborated 5 years later when Naguib et al. (1985), measuring the *in vitro* kinetics of the enzymes of FUra catabolism, determined the $K_M$ of human dihydropyrimidine dehydrogenase (DPD) for FUra to be 14 μM. For these reasons, a fixed $K_M$ of 15 μM was used for this study.

### *Model application – effect of interferon*

Each patient's data were fitted to derive values for volume of distribution ($V_d$), maximum non-linear elimination rate ($V_{max}$) and first-order elimination rate constant ($k_{1,e}$). With the exception of the two excluded outlier patients, good fits were obtained with individual patient data during both bolus and infusion phases, with weighted regression coefficients in the range $r^2 = 0.985–0.998$.

The comparison of FUra/LV alone and FUra/LV/IFN-α patients is shown in Table II. The Shapiro–Wilk test shows that each variable is normally distributed, so parametric statistics have been used. No statistically significant differences are found between the two patient groups. The widest difference in mean values occurs for $V_{max}$, but inter-patient variability is greatest for this parameter, so the power to detect a difference is reduced. Thus the data are not inconsistent with a reduction of up to 48% in $V_{max}$ after IFN-α treatment. On the other hand, $V_d$ and $k_{1,e}$ show little inter-patient variability, and the data are able to exclude a true

**Table II** FUra pharmacokinetic parameters in patients receiving FUra/LV or FUra/LV/IFN-α

| | *FUra/LV control (n = 9)* | | | *FUra/LV/IFN-α (n = 9)* | | | | *95% CI for* |
|---|---|---|---|---|---|---|---|---|
| | *Mean* | *s.d.* | *Coefficient of variation (%)* | *Mean* | *s.d.* | *Coefficient of variation (%)* | *P-value IFN-α vs control* | *IFN-α/control ratio* |
| $AUC_{0-22}$ (μM h$^{-1}$) | **115.7** | 24.8 | 21 | **125.2** | 18.9 | 15 | 0.38 | 0.89–1.27 |
| $V_d$ (l m$^{-2}$) | **10.39** | 1.89 | 18 | **10.00** | 0.94 | 9 | 0.58 | 0.81–1.11 |
| $V_{max}$ (μM h$^{-1}$) | **181.8** | 58.7 | 32 | **141.3** | 26.7 | 19 | 0.09 | 0.52–1.04 |
| $k_{linear}$ (h$^{-1}$) | **4.352** | 0.575 | 13 | **3.961** | 0.513 | 13 | 0.15 | 0.78–1.04 |

**Table III** Effect of IFN-α on FUra kinetics – previous studies

| *Reference* | *FUra schedule* | n | *LV* | *IFN-α dose (corr. 1.7 m²)* | *Significant effect on FUra pharmacokinetics* |
|---|---|---|---|---|---|
| Grem *et al.* (1991) | bolus | 6 | + | 5 MU o.d. | – |
| | | 6 | + | 8.5 MU o.d. | – |
| | | 6 | + | 17 MU o.d. | + |
| Schuller *et al.* (1992) | bolus | 10 | – | 5 MU t.i.w. | + |
| | | 10 | + | 5 MU t.i.w. | – |
| Kreuser *et al.* (1992) | 4 h inf. | 10 | + | 5 MU o.d. | – |
| Danhauser *et al.* (1993) | 120 h inf. | 21 | – | 0.2–25 MU o.d. | + |
| Pittman *et al.* (1993) | 120 h inf. | 26 | – | 9 MU t.i.w. | – |
| Lindley *et al.* (1990) | PI | 9 | – | 3.5–10 MU o.d. | + |
| Sparano *et al.* (1993) | PI | 26 | – | 8.5 MU t.i.w. | – |

PI, protracted ambulatory infusion.

difference of more than 18% and 22% respectively in these two parameters. Using partial area predictions, the linear elimination route accounts for >90% of the total drug clearance during the initial hour after FUra bolus. However, the non-linear route accounts for >55% of total clearance during the steady-state infusion.

### *Organ function*

Two-step regression was used to assess the effect of organ function on each of the pharmacokinetic parameters after correction for any effect of interferon. No significant correlations were found for serum alkaline phosphatase, serum aspartate transaminase or estimated creatinine clearance (using the formula of Cockcroft & Gault, 1976).

### Discussion

We have not been able to demonstrate any effect of IFN-α, at 6 MU on alternate days, upon the pharmacokinetics of FUra. The power of the study is, of course, limited by its size; however, we are able to exclude any effect which is large in comparison with inter-patient variability. This finding is contrary to some, but not all, previous reports.

The bolus-plus-infusion FUra schedule used in this study has permitted the development of a relatively simple mathematical model for the disposition of FUra over a wide range of plasma concentrations. As a purely mathematical tool, its elements do not directly represent physiological drug disposition processes. For example, in this model the non-linear route accounts for only a minority of FUra elimination during the bolus phase, although Coustère *et al.* (1991) have shown, using plasma, urinary and biliary catabolite modelling, that the dihydropyrimidine dehydrogenase (DPD) catabolic pathway is responsible for 55% of FUra clearance after a 500 mg m$^{-2}$ bolus injection.

By contrast, Collins *et al.* (1980) described an operational model, with linear elimination set at the glomerular filtration rate (representing renal clearance of unchanged drug) and a Michaelis–Menten route representing drug metabolism. Applying a similar approach to the current data, a fit is obtained for most patients only when a very capacious peripheral compartment is introduced, with $k_{12} >> k_{21}$: in practice, an additional linear elimination pathway.

With these reservations about the interpretation of parameters in the mathematical model used in this study, it might nonetheless be expected that changes in DPD activity would be primarily reflected as changes in the $V_{max}$. In a recent study, Yee *et al.* (1992) found that DPD activity in patients' peripheral blood mononuclear cells fell by 50% after 4 days of IFN-α treatment. In the current study, no statistically significant difference in $V_{max}$ was found, although the wide variability of this parameter means that a reduction of up to 48% could have been missed.

Previous studies of FUra/IFN-α pharmacokinetic interaction have yielded inconsistent results (see Table III). All have used a paired cycle design, measuring plasma levels during FUra (or FUra/LV) treatment, then adding IFN-α and re-sampling. This has the advantage of correcting for inter-patient variability, but may introduce systematic error when treatments are always given in the same order. The studies have involved either bolus or infusional regimens, not both, and have assumed linear kinetics, deriving values for *apparent* plasma clearance (Cl) and *apparent* half life.

Two studies involve bolus FUra regimens. Grem *et al.* (1991) measured the kinetics of bolus FUra (with high-dose LV) before and after IFN-α2a at 3, 5 and 10 MU m$^{-2}$ day$^{-1}$, and reported a significant reduction of 25% in FUra clearance at the highest IFN-α dose. The results of Schüller *et al.* (1992) are provocative: bolus FUra kinetics was measured in ten patients, first on FUra alone, then on FUra with IFN-α2b and finally on triple therapy including high-dose LV. A linear two-compartment model was applied, and the authors found significantly decreased mean FUra clearance and increased mean AUC for FUra/IFN-α compared with FUra alone. These changes appeared to be reversed when LV was introduced, leading to postulation that IFN-α decreases FUra's metabolic clearance and that its effect is negated by leucovorin. However, the inter-patient variability was greater for FUra/IFN-α than for the other data sets, with three patients having end-of-bolus plasma levels 2–3 times greater than the median. In the presence of non-linearity, this may have produced artefactually low values for FUra 'clearance': the conclusion of the study may have been different had an appropriate non-linear model been used.

Turning to infusional regimens, an early abstract report of dramatic elevation of FUra levels within 1 h of administering IFN-α2b has yet to be published in full (Lindley *et al.*, 1990). More recently Danhauser *et al.* (1993), assuming that steady-state FUra plasma concentration had been reached after 50 min (five 'half-lives') of FUra continuous infusion, calculated $Cl_{ss}$ from a single sample then introduced IFN-α2b after 16–21 h. There was a statistically significant decrease in

$Cl_{ss}$ after adding IFN-α; however the complete lack of IFN-α dose relationship over the range 0.1–15.0 MU $m^{-2}$ suggests that the difference may have been caused by the timing of samples rather than by IFN-α. Sparano *et al.* (1993) made a more thorough assessment of steady-state kinetics, using at least seven samples over 48 h, before introducing IFN-α2a. Care was taken to correct for diurnal variation in FUra kinetics by performing cosinor analysis before and after IFN-α addition. No effect of IFN-α on FUra kinetics was seen. Similarly, Pittman *et al.* (1993), in the only study to use a random-order crossover design, found no effect of IFN-α2a on $[FUra]_{ss}$ in 26 patients. Kreuser *et al.* (1992), also using multiple samples, measured drug levels during a 4 h infusion of 500 mg $m^{-2}$ FUra before and after the addition of IFN-α2b: again, no difference was found.

In conclusion IFN-α, at 6 MU on alternate days, has no significant effect on the pharmacokinetics of FUra. The current study has the advantages of examining both bolus and infusional phases of FUra administration, and of using a non-linear pharmacokinetic model which avoids the artefactual variations in apparent clearance which arise with the inappropriate use of linear modelling. Review of the literature suggests that, at higher doses of IFN-α around 10 MU $m^{-2}$ $day^{-1}$, FUra elimination may be reduced, probably through decreased DPD activity. However, some of the studies which have suggested an effect should be interpreted with reservation, and there is no conclusive published evidence for a pharmacokinetic interaction at lower doses of IFN-α.

### References

CHRISTOPHIDIS, N., MIHALY, G., VAJDA, F. & LOUIS, W. (1979). Comparison of liquid and gas-liquid chromatographic assays of 5-fluorouracil in plasma. *Clin. Chem.*, **25**, 83–86.

CHU, E., ZINN, S., BOARMAN, D. & ALLEGRA, C.J. (1990). Interaction of γ interferon and 5-fluorouracil in the H630 human colon carcinoma cell line. *Cancer Res.*, **50**, 5834–5840.

COCKCROFT, D.W. & GAULT, M.H. (1976). Prediction of creatinine clearance from serum creatinine. *Nephron*, **16**, 31–41.

COLLINS, J.M., DEDRICK, R.L., KING, F.G., SPEYER, J.L. & MYERS, C.E. (1980). Non-linear pharmacokinetic models for 5-fluorouracil in man: intravenous and intraperitoneal routes. *Clin. Pharm. Ther.*, **28**, 235–246.

COUSTÈRE, C., MENTRÉ, F., SOMMADOSSI, J.-P., DIASIO, R.B. & STEIMER, J.-L. (1991). A mathematical model of the kinetics of 5-fluorouracil and its metabolites in cancer patients. *Cancer Chemother. Pharmacol.*, **28**, 123–129.

DANHAUSER, L.L., FREIMANN, J.H., GILCHRIST, T.L., GUTTERMAN, J.U., HUNTER, C.Y., YEOMANS, A.C. & MARKOWITZ, A.B. (1993). Phase I and plasma pharmacokinetic study of infusional fluorouracil combined with recombinant interferon alpha-2b in patients with advanced cancer. *J. Clin. Oncol.*, **11**, 751–761.

DE GRAMONT, A., KRULIK, M., CADY, J., LAGADEC, B., MAISANI, J.-E., LOISEAU, J.-P., GRANGE, K.-D., GONZALEZ-CANALI, G., DEMUYNCK, B., LOUVET, C., SEROKA, J., DRAY, C. & DEBRAY, J. (1988). High-dose leucovorin and 5-fluorouracil bolus and infusion in advanced colorectal cancer. *Eur. J. Clin. Oncol.*, **24**, 1499–1503.

GREM, J.L., MCATEE, N., MURPHY, R.F., BALIS, F.M., STEINBERG, S.M. HAMILTON, J.M., SORENSEN, M., SARTOR, O., KRAMER, B.S., GOLDSTEIN, L.J., GAY, L.M., KAUBO, K.M., GOLDSPIEL, B. & ALLEGRA, C.J. (1991). A pilot study of interferon α2a in combination with fluorouracil plus high-dose leucovorin in metastatic gastrointestinal carcinoma. *J. Clin. Oncol.*, **9**, 1811–1820.

HEINZEL, G., WOLOSZCZAK, R. & THOMANN, P. (1993). *TopFit Version 2.0, Pharmacokinetic and Pharmacodynamic Data Analysis System for the PC.* Dr. Karl Thomae: Stuttgart; G. Fischer: New York.

HOUGHTON, J.A., ADKINS, D.A., RAHMAN, A. & HOUGHTON, P.J. (1991). Interaction between 5-fluorouracil, [6RS] leucovorin, and recombinant human interferon-α2a in cultured colon adenocarcinoma cells. *Cancer Commun.*, **3**, 225–231.

HOUGHTON, H.J., MORTON, C.L., ADKINS, D.A. & RAHMAN, A. (1993). Locus of the interaction among 5-fluorouracil, leucovorin, and interferon-α2a in colon carcinoma cells. *Cancer Res.*, **53**, 4243–4250.

KREUSER, E.D., HILGENFELD, R.U., MATTHIAS, M., HOKSCH, B., BOEWER, C., OLDEN-KOTT, B., KNAUF, W.U., BOESE-LANDGRAF, J., SCHALHORN, A., ZEITZ, M. & THEIL, E. (1992). A phase I trial of interferon-α2b with folinic acid and 5-fluorouracil administered by 4-hour infusion in metastatic colorectal carcinoma. *Semin. Oncol.*, **19** (Suppl. 3), 197–203.

LINDLEY, C., BERNARD, S., GAVIGAN, M., KALUZNY, B. & MEADOWS, L. (1990). Interferon-alpha increases 5-fluorouracil levels 64-fold within 1 hour: results of a phase I study (abstract II 6–31). *J. Interferon Res.*, **10** (Suppl. 1), S132.

NAGUIB, F.N.M., EL KOUNI, M.H. & CHA, S. (1985). Enzymes of uracil catabolism in normal and neoplastic human tissues. *Cancer Res.*, **45**, 5405–5412.

PITTMANN, K., PERREN, T., WARD, U., PRIMROSE, J., SLEVIN, M., PATEL, N. & SELBY, P. (1993). Pharmacokinetics of 5-fluorouracil in colorectal cancer patients receiving interferon. *Ann. Oncol.*, **4**, 515–516.

SCHULLER, J., CZEJKA, M.J., SCHERNTHANER, G., FOGL, U., JAGER, W. & MICKSCHE, M. (1992). Influence of interferon α2b with or without folinic acid on pharmacokinetics of fluorouracil. *Semin. Oncol.*, **19** (Suppl. 3), 93–97.

SCHWARTZ, E.L., HOFFMAN, M., O'CONNOR, C.J. & WADLER, S. (1992). Stimulation of 5-fluorouracil metabolic activation by interferon-α in human colon carcinoma cells. *Biochem. Biophys. Res. Commun.*, **182**, 1232–1239.

SEYMOUR, M.T., DOBSON, N., CLEMENS, M.J. & SLEVIN, M.L. (1992). 5-Fluorouracil/interferon-α synergy: is regulation of expression of thymidylate synthase the key? *Proc. Annu. Meet. Am. Assoc. Cancer Res.*, **33**, 545.

SPARANO, J.A., WADLER, S., DIASIO, R.B., ZHANG, R., LU, Z., SCHWARTZ, E.L., EINZIG, A. & WIERNIK, P.H. (1993). Phase I trial of low-dose, prolonged continuous infusion fluorouracil plus interferon-alfa: evidence for enhanced fluorouracil toxicity without pharmacokinetic perturbation. *J. Clin. Oncol.*, **11**, 1609–1617.

WADLER, S. & SCHWARTZ, E.L. (1990). Antineoplastic activity of the combination of interferon and cytotoxic agents against experimental and human malignancies: a review. *Cancer Res.*, **50**, 3473–3486.

WADLER, S. & WIERNIK, P.H. (1990). Clinical update on the role of fluorouracil and recombinant interferon-α2a in the treatment of colorectal carcinoma. *Semin. Oncol.*, **17** (Suppl. 1), 6–21.

YAMAOKA, K., NAKAGAWA, T. & UNO, T. (1978). Application of Akaike's Information Criterion in the evaluation of linear pharmacokinetic equations. *J. Pharmacokin. Biopharm.*, **6**, 165–175.

YEE, L.K., ALLEGRA, C.J., STEINBERG, S.M. & GREM, J.L. (1992). Decreased catabolism of fluorouracil in peripheral blood mononuclear cells during combination chemotherapy with fluorouracil, leucovorin and interferon-α2a. *J. Natl Cancer Inst.*, **84**, 1820–1825.